# Beer and Microbiota: Pathways for a Positive and Healthy Interaction

**DOI:** 10.3390/nu15040844

**Published:** 2023-02-07

**Authors:** Corina-Aurelia Zugravu, Cosmin Medar, Loredana Sabina Cornelia Manolescu, Ciprian Constantin

**Affiliations:** 1 Hygiene and Nutrition Discipline, Department of Fundamental Sciences, Faculty of Midwifery and Nursing, “Carol Davila” University of Medicine and Pharmacy, 020021 Bucharest, Romania; 2 National Center for Monitoring the Community Risks, Institute of Public Health, 050463 Bucharest, Romania; 3 Patient Management in the Radiology and Imaging Service, Radiology Techniques Discipline, Department of Special Sciences, Faculty of Midwifery and Nursing, “Carol Davila” University of Medicine and Pharmacy, 050474 Bucharest, Romania; 4 Microbiology, Parasitology and Virology Discipline, Department of Fundamental Sciences, Faculty of Midwifery and Nursing, “Carol Davila” University of Medicine and Pharmacy, 020021 Bucharest, Romania; 5 Department of Virology, “Stefan S. Nicolau”, Institute of Virology, 030304 Bucharest, Romania; 6 Research Metabolic Centre, 011055 Bucharest, Romania; 7 Carol Davila Emergency Military Hospital, 010817 Bucharest, Romania

**Keywords:** beer, microbiota, polyphenols, dietary fibers, melanoidins

## Abstract

Beer is one of the most consumed drinks worldwide. It contains numerous categories of antioxidants, phenolic products, traces of group B vitamins, minerals (selenium, silicon, potassium), soluble fibers and microorganisms. Low or moderate beer consumption, with or without alcohol, showed positive effects on health by stimulating the development of a healthy microbiota. In the present review we focused on four components responsible with interaction with gut microbiota: microorganisms, polyphenols, fiber and melanoidins, their presence in usual beers and on perspectives of development of fortified beers with enhanced effects on gut microbiota. Though microorganisms rarely escape pasteurization of beer, there are new unpasteurized types that might bring strains with probiotic effects. The polyphenols from beer are active on the gut microbiota stimulating its development, with consequent local anti-inflammatory and antioxidant effects. Their degradation products have prebiotic action and may combat intestinal dysbiosis. Beer contains dietary fiber such as non-starchy, non-digestible carbohydrates (β-glucans, arabinoxylans, mannose, fructose polymers, etc.) that relate with gut microbiota through fermentation, serving as a nutrient substrate. Another type of substances that are often considered close to fiber because they have an extremely low digestibility, melanoidins (melanosaccharides), give beer antioxidant and antibacterial properties. Though there are not many research studies in this area, the conclusion of this review is that beer seems a good candidate for a future functional food and that there are many pathways by which its ingredients can influence in a positive manner the human gut microbiota. Of course, there are many technological hinderances to overcome. However, designing functional beers fortified with fiber, antioxidants and probiotics, with a very low or no alcoholic content, will counteract the negative perception of beer consumption, will nullify the negative effects of alcohol, while simultaneously exerting a positive action on the gut microbiota.

## 1. Introduction

One of the most consumed drinks on the whole world is represented by beer. With a consumption history of several millennia, proven by very old archaeological discoveries, having a manufacturing technology that has evolved over time to the current form of the drink, it can have a significant impact on the health of people and the effects can be major, due to the enormous area where beer is drunk. In the past, beer also had medicinal values, as a stimulant or analgesic [1]. Beer was already widely consumed in ancient Egypt [2]. If until recently the history of beer went all the way to Babylonia, around 6000 BC, today there is evidence that a product similar to beer was consumed in China 9000 years ago, in a ritualic context [3]. Like other alcoholic beverages, beer is viewed with caution in the medical world. However, similar to other fermented products, it has health potential and beers without alcohol can be easily produced. It contains numerous categories of antioxidants, especially phenolic products from both hops and malt [4,5], traces of vitamins (especially from group B) [6] and minerals (selenium, silicon, potassium) [6] and soluble fibers [7]. Some bacteria and fungi have also been identified in the beer, the most encountered being lactic acid bacteria (*Lactobacillales* order, *Bifidobacterium* genus) and *Saccharomyces* spp. [8,9,10].

Alcohol itself can have dual effects, depending, of course, on the amount consumed. The composition, as well as the versatility of the manufacturing process that allows obtaining beer with minimal or no alcohol, identifies it as a potential functional food as such, but above all it can become functional through the addition of biologically active substances. We exclude excessive consumption of alcoholic beer from the start, due to harmful effects. Numerous studies have followed the action of low or moderate beer consumption, with and without alcohol, on health. The identified effects were positive in the following directions: cardio-protective effect [11], bone health [12], positive stimulation of microbiota [13], etc. In the entire evaluation of beer, the current consumption trends must be taken into account, in which the individual is interested in the health benefits of the products consumed, beyond the satisfaction and covering of basic survival needs. The highlighting of the natural presence of some sanogenic ingredients or of the enrichment of the product in a specific ingredient with positive action on health can be beneficial for both the producer and the consumer [14]. 

Microbiota, the living microorganisms from oral cavity, gut, skin and other sites such as vagina or lungs was studied by numerous researchers since it was discovered back in early 1900s [15]. It harbors a whole range of microorganisms, which from the most studied are bacteria and fungi that are symbiotic with the human body generating its optimal functioning [16,17,18,19], Table 1.

In the human microbiota there are also Archaea and Viruses which roles are not yet completely understood [20]. The gut microbiota, composed of more than 100 trillion microorganisms [21], is the most studied, and several roles such as food fermentation, production of vitamins or even immune roles have been attributed to it. In humans, gut microbiota differs at the individual level, by localization into the gastro-intestinal tract and by age [22]. There are different conditions (pH and level of oxygen) that induce colonization with bacterial types: in the small intestine *Proteobacteria (Enterobacteriaceae)* are found and in the colon *Bacteriodetes (Bacteroidaceae, Prevotellaceae and Rikenellaceae)* were detected [23]. Gut microbiota becomes relatively stable from the age of three [24], but in people over seventy its diversity changes, with low levels of *Bifidobacterium* and high levels of *Clostridium* and *Proteobacteria* [25]. The alteration of healthy gut microbiota can determine an unbalanced composition of bacterial population, leading to various diseases such as cardiovascular diseases, cancer, diabetes mellitus, inflammatory bowel diseases, chronic liver diseases and chronic kidney diseases [26].

**Figure 1:**
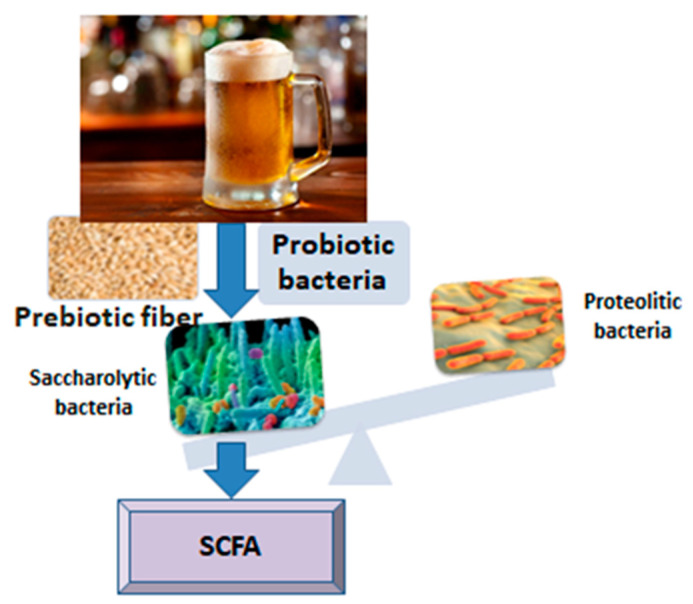
From beer to short chain fatty acids (SCFA).

The relationship between beer or some of its ingredients and the intestinal microbiota is interesting and has been revealed in several studies to date, offering generous premises for future research. The influence of food ingredients on the composition, diversity and functionality of the intestinal microbiota is still incompletely deciphered, especially when it comes to lasting effects over time (Figure 1). It should be noted that chronic alcohol consumption has negative effects on the diversity of the microbiota, causing intestinal dysbiosis [13], among other ill effects on human metabolism. In the present narrative review, we targeted four components of beer that might interact with gut microbiota, according to research: microorganisms, antioxidants, fiber and melanoidins. By means of pre and probiotic mechanisms, they show potential to enhance the development of a healthy gut microbiota, with predominant saccharolytic, short chain fatty acids-producing bacteria.

## 2. Methodology

Major databases (PubMed, Scopus, Web of Science, Google Scholar) were searched for items followed in this review and results are presented in Table 2.

## 3. Beer and its Principal Interactions with the Microbiota

### 3.1. The Microorganisms from Beer and Their Probiotic Potential

Beer is usually a pasteurized product, but there are crafted beers that have a potential of influencing the gut microbiota because they contain bacteria. Studies of the effect of beer consumption on the gut microbiota are few. A study from 2019 detected by sequencing of 16S rDNA eighteen genera of bacteria present in the rice beer, *Lactobacillus* being the dominant group (90%). Other types were *Acetobacter*, *Acinetobacter*, *Bacillus*, *Dickeya*, *Enterococcus*, *Enterobacter*, *Exiguobacterium*, *Gluconobacter*, *Janibacteria*, *Klebsiella*, *Lactococcus*, *Leuconostoc*, *Pseudomonas*, *Pediococcus*, *Rothia*, *Staphylococcus* and *Weissella*. Based on the detected bacteria and their bacterial profiles metabolic pathways were revealed as being influenced by the consumption of the rice beer, such as metabolisms of carbohydrate, amino acid, vitamins and cofactors, as well as xenobiotic biodegradation [89]. 

The main effect of alcohol intake on gut microbiota is dysbiosis [13,31], by changing the balance of the dominant bacterial from *Phyla Bacteroidetes*, *Firmicutes* and *Phylum* proteobacteria. However, this is not the case with usual beer, when consumed with moderation, because it contains about 5% alcohol. Low-alcohol and alcohol-free beers are popular and widely consumed. So, when considering beer, it is important to choose a type of beer with low or without alcohol, which gives the benefits of fermented foods.

Beer enriched with Saccharomyces cerevisiae strain intake may modulate gut microbiota and have beneficial influence the symptoms in Alzheimer’s disease, generating a neuroprotective effect by ameliorating cognition and increasing the concentration of anti-inflammatory cytokines, as new data revealed in 2022 [9]. Another type of beer rich in bacterial composition is Belgian lambic beer. The different bacteria present throughout the production process result because of the spontaneous inoculation of microorganisms from the environmental air and the inner surfaces of the wooden barrels [86]. Several new bacterial species such as *Acetobacter lambici* and *Gluconobacter cerevisiae* have been described in lambic beer. In the process of production of this type of beer, the following bacteria are present: Enterobacteria (*Enterobacter cloacae*; *Klebsiella oxitoca*), acetic acid bacteria (*Acetobacter* spp.; *Gluconobacter cerevisiae*) and lactic acid bacteria (*Pediacoccus* spp.), along with different yeasts (*Hanseniaspora uvarum*; *Saccharomyces* spp.; *Brettanomyces* spp.), with possible, not-yet-studied influence on gut microbiota. Crafted beers with several enhanced tastes, such as fruits, herbs, honey, spices and vegetables, have become more popular lately, but because they are not always pasteurized or sterilized by filtration they are subject to spoilage, due to the microbiota associated with the organic raw ingredients added to obtain the special tastes. Even if there is not enough knowledge available about the microbiota diversity in craft breweries it is known that some lactic acid bacteria from beer can produce biogenic amines such as histamine, tyrosine, putrescine and cadaverine, which can alter the beer and have possible toxic effects. Biogenic amines can also be found in sausages, fermented vegetables, fishery products, cheese and wine [32]. Published studies of sixty monitored points inside the craft brewery revealed that *Lactobacillus*, *Pediococcus* and *Leuconostoc genera*, are responsible for biogenic amines production, especially two isolates of *Lactobacillus brevis* that are able to be cultured into acidic conditions, with more hop and alcohol, these isolates had presented horA, horC and hitA genes, and the highest production of biogenic amines [90]. Corn beer has potential sources of probiotic lactic acid bacteria with cholesterol lowering activity, the strains identified by sequencing the 16S rRNA gene were *Levilactobacillus brevis* and *Enterococcus faeccium*, NCBI genbank accension numbers ON454506 and ON908682; isolates that effectively lowered LDL-c and increased HDL-c in rat sera, which are the main risk factors for cardiovascular diseases [84]. Beer is a fermented beverage that has enhanced nutritional and functional properties due to transformation of substrates, formation of bioactive end-products and presence of living microorganisms, genetically similar to strains used as probiotics [27]. Some studies revealed that beer components may have antimicrobial properties, as well as microbiological spoilage risks [91]. 

The microbial community (bacteria and fungi) from beer differs in time because it is influenced by its initial composition, the quantity of alcohol and the type of barrel where it is kept. Studies that used amplicon sequencing of the V4 region of the bacterial 16S rRNA gene and the fungal ITS1 region have shown using PerMANOVA analysis that during the process of beer maturation significant higher levels in the bacterial and fungal population appeared. The lactic acid bacteria became dominant in the moderately hopped beers and remained fairly constant in high-bitterness beer; with similar composition of the traditional beers, *Pediococcus damnosus*, *Lactobacillus brevis* and *Acetobacter* spp., and the fungi were influenced by the presence of alcohol [85]. There are even studies that introduced the idea of non-*Saccharomyces* yeast beers, and these types of beers may enter on the market in the future, after investigations and guideline for the safety assessment of yeasts are carried out [92].

So, bacteria and fungi encountered in the beer fabrication process, enriched or crafted beer, produce an array of compounds such as vitamins, bacteriocins and organic acids which confer health benefits to the consumers and can modulate the indigenous intestinal flora of the host.

### 3.2. Polyphenols and Microbiota

**Figure 2:**
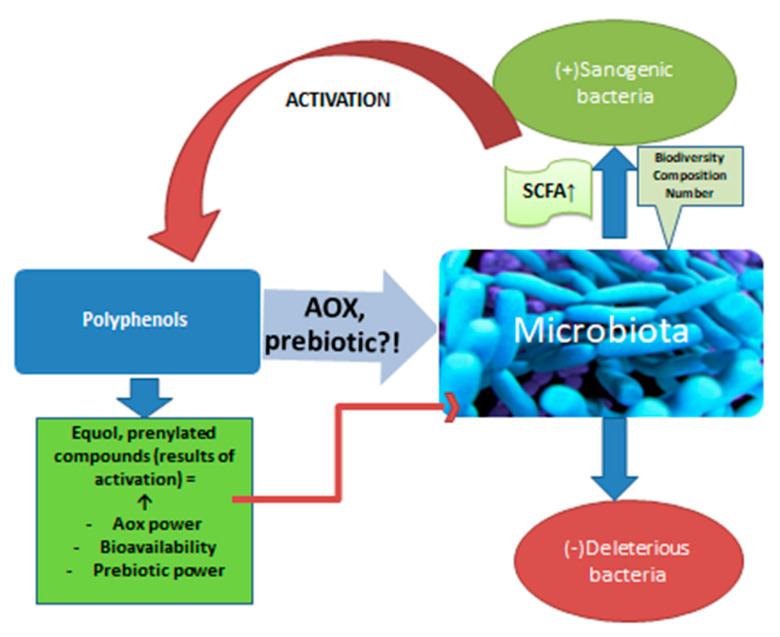
The action of polyphenols on the microbiota.

Beer is an important vehicle for polyphenols, which, together with bitter acids, form beer’s antioxidants. Most of them come from malt and only about 20% from hops. There are in vitro studies that confirm the action of polyphenols on the microbiota [93] (Figure 2). Animal studies support this interaction. For example, a polyphenol well represented in beer, ferulic acid, amplifies the biodiversity of the microbiota and stimulates the multiplication of bacteria that produce propionate and butyrate in the colon of rats [36]. Although limited in number, studies on human subjects also confirm the interaction under discussion. Studies show that after the ingestion of polyphenols, the production of short chain fatty acids (SCFA) increases, with consequent local anti-inflammatory effects [37]. The increase in SCFA synthesis confirms the action on the microbiota, an effect already demonstrated in the case of red wine consumption, which is itself a source of polyphenols. Red wine increased the levels of *Bifidobacterium* in the microbiota, as well as *Faecalibacterium prausnitzii* and *Roeburia*. The development of *Enterobacter* or *Escherichia coli* strains was inhibited [38]. These positive effects were also observed for alcohol-free wine. So, although the absorption of polyphenols from alcohol-free products (beer, wine) decreases compared to the same products with alcohol [4,94], polyphenols not absorbed that reach the colon have important effects in situ with multiple repercussions through the action on the intestinal microbiota. It is about oligo and polymeric polyphenols that usually do not undergo transformations until the distal intestine [37]. Polyphenols are “activated” by certain populations of the microbiota, especially when it comes to phytoestrogens [39,40,95]. Polyphenols are transformed by bacteria into absorbable products that reach through the portal blood, to the liver or into prenylated products that have important sanogenic actions, such as the antiproliferative action on some cell lines, as prenyl naringenin and xanthohumol have [41]. The interrelation between polyphenols and microbiota is extremely complex. The microbiota increases the bioavailability of polyphenols which, in turn, modulate the composition of the populations in the colon, inhibiting pathogenic microorganisms and stimulating the development of healthy ones through a prebiotic action [42,43]. Quercitin, a flavonoid from beer, combated intestinal dysbiosis, improving the ratio between Firmicutes and Bacteroides populations and opposing the proliferation of microbiota species associated with excessive body weight [48,49]. It should be noted that by drinking non-alcoholic beer or wine, you not only avoid the negative effects determined by ethyl alcohol, including on the microbiota, but also increase the number of polyphenols that reach the intestine and which would have positive effects on the microbiota.

In a study conducted by Hernández-Quiroz et al. [46], after the administration of a dose of 355 mL of beer per day for 30 days in healthy subjects, divided into a group that received beer without alcohol (*n* = 35) and one that received beer with alcohol (*n* = 33), a clear influence on the intestinal microbiota, as well as on the functionality of pancreatic β cells and fasting blood glucose, could be observed. The action on the microbiota consisted of increasing the diversity of the microbiota, by favoring species of the *Bacteroides* type, at the expense of *Firmicutes*. The authors attribute this effect to the polyphenols in beer, and it was found in beer without alcohol. Beer with alcohol did not have positive effects of the same scope and negatively impacted blood sugar and β cell functionality. 

Another observational study [47] found an increase in butyric acid, a byproduct of the intestinal microbiota, in beer consumers, but without quantifying the intake of polyphenols. Positive effects were found only with wine in a large study on twins in Great Britain, in which non-alcoholic beer was not an element of investigation [96]. A recent clinical study by Martínez-Montoro et al. [97] worked on adults aged 30–60, divided into two groups (with or without metabolic syndrome) who were administered beer with different concentrations of polyphenols, consumed successively, after respective washout periods. In the beginning there were no radical differences in microbiota characteristics between the two groups. During the study, the authors report significant changes in the microbiota, with substantial changes in the entire profile, all the more important as the polyphenol content of the beer was higher. The changes were also influenced by the metabolic status of the individuals, being significant in the group of subjects with metabolic syndrome, where the abundance of streptococcus was highest after consumption of dark beer. A previous study showed that certain species of streptococci interact with gallic acid and catechins, amplifying their antioxidant effects [48]. Moreover, some streptococci can transform beer melanoidins into an isoflavone with estrogenic and antioxidant action called equol [49]. The authors attribute the effects found in the group with metabolic syndrome to the correction of intestinal dysbiosis, which is usually present in individuals with the syndrome in question. Since the most important changes were found after the consumption of dark beer, very rich in antioxidant polyphenols, the authors explain the influence also through the respective antioxidant action on the microbiota, excluding the possible interference of alcohol, which was found in equal quantities in lager beer (with fewer polyphenols) and in the black one (with maximum level of polyphenols).

There are still open study perspectives in which to possibly follow the impact of some types of beer enriched in polyphenols, with or without alcohol, on the intestinal microbiota and from here, on the entire metabolism [4]. An extensive review of the effects of beer polyphenols on the microbiota was carried out by Quesada-Molina et al. [50]. The authors analyze the existing studies, noting that in principle a detailed analysis of the microbiota-polyphenols interaction is needed and that the existing results so far are based on deduction rather than on concrete quantification of the effects.

### 3.3. Dietary Fibers in Beer and Gut Microbiota

In the tables of data on the composition of foods, beer is not mentioned as containing dietary fiber. In reality, beer contains a series of non-starchy, non-digestible carbohydrates, such as β-glucans (approximately 75%), arabinoxylans (approximately 20%), arabinogalactans and their fragments, mannose and fructose polymers as well as resistant starch [50,54,55]. Detailed tests performed on various types of beer showed the existence of arabinoxylan derivatives with a medium to high degree of polymerization and β-glucans with a significantly lower degree of polymerization. Numerous small oligosaccharides of β-glucosyl or pentosyl type (arabinoxylans with reduced polymerization) have also been identified as being present in a rather high quantity [56].

Scientists are working to develop analytical methods to quantify these compounds [57]. In this context, it is discussed to what extent the presence of soluble fiber in beer has or does not have an impact on the health of consumers through the intestinal microbiota [50]. Trials undertaken to date place the level of dietary fiber in beer in the range of 0.5–4 g/L [54], varying depending on the technological solutions applied and the assortment, more precisely, depending on the content in the wort extract [58]. The respective quantities are small compared to the quantity required to be able to use a nutritional claim on beer labels. Reid et al. [59], citing Li and Du [56], approximates the amounts of non-starchy carbohydrates as 0–1.5 g L^−1^ in blonde beers such as lager, 1.0–2.0 g L^−1^ in brown beers, the highest amount being in wheat beers (1.5–4.0 g L^−1^).

Recent studies argue that beer, including alcohol-free or low-alcohol beer, could contribute substantially to the intake of soluble fibers [56,57,59].

The connection of dietary fibers with the intestinal microbiota is very close and is related to the fermentability of the fibers. It is about their characteristic of serving as a nutrient substrate for the bacteria of the intestinal microbiota. Many of the dietary fibers have prebiotic action. Prebiotics are defined as “dietary ingredients selectively metabolized by certain phylogenetic groups of the intestinal microbiota, which cause specific changes—both composition and activity—of the human microbiome, conferring physiological and health benefits” [60]. The compounds considered to have prebiotic activity are of a carbohydrate nature, and, most often, they are from the category of inulins, fructo-oligosaccharides, galacto-oligosaccharides, soy-oligosaccharides, xylo-oligosaccharides, pyrodextrins, isomalto-oligosaccharides or lactulose [61]. Not every type of fiber is a prebiotic. In order to have an effect, prebiotics must keep their structure unmodified under the action of various factors that act along the digestive tract up to the level of the colon. It should be noted that because they are not altered by the enzymes of the human digestive system, prebiotics are considered soluble dietary fibers [62], but there are also dietary fibers without prebiotic action or prebiotic substances that are not fibers according to their classical definition. An indicator often used to monitor the effect on the microbiota is the production of SCFA through the fermentation of prebiotics, which are a source of energy for intestinal anaerobes and have various sanogenetic effects, from increasing the absorption of nutrients, to balancing glucose metabolism, stimulating immunity, influencing lipid metabolism, etc.

As stated about, beer contains some fiber; however, research has usually targeted effects of fiber found in beer, not beer by itself. For several years, an increased interest has been aroused by the prebiotic effect of the hydrolysis products of prebiotic fiber, i.e., arabinoxylo-oligosaccharides(AXOS), xylo-oligosaccharides (XOS) and β-glucano-oligosaccharides (βGOS). There are already available on the market ingredients with a high content of AXOS, XOS or βGOS, intended for the enrichment/fortification of bakery products or other cereal products, even beer, but only for the purpose of changing the palatability [33]. Generally, β-glucans from barley are already hydrolyzed during malting and mashing, in the process of beer fabrication, resulting in low molecular weight oligosaccharides (βGOS) that cannot be digested by *S. cerevisiae*. An in vitro study tested the ability of βGOS as a prebiotic [50] and showed that the substance can survive in conditions of pH and enzymatic stress similar with those in a human digestive tract. Different strains of *Lactobacillus* were able to survive by only using b GOS as source of carbon. Authors calculate that with a daily intake of 0.33 l of beer providing 4 mg/mL β-GOS [56], the quantity reaching the colon would be 1.34 g/L, which is far below the quantities used in the study. Other in vitro tests using colon models indicated a considerable prebiotic potential of oligosaccharides derived from β-glucans and pentosans from barley, wheat and oats. A study designed to compare the prebiotic activity of hydrolyzed β-glucan derivatives reveals a significant stimulating effect on the populations of the *Lactobacillus-Enterococcus* group for βGOS from barley with 3–4 monomer units, in contrast to non-hydrolyzed β-glucan fibers, which marginally favored the development these bacteria, but less compared to inulin [63]. XOS from barley stimulated the accumulation of short-chain fatty acids—especially butyrate—in the vessels of the colon model and demonstrated a greater ability to stimulate the growth of species of the *Bifidobacterium lactis* group compared to FOS and inulin, favoring *B. longum* [64]. Perhaps more than in the case of other fibers, the action of β-glucans on the microbiota is well described. Their origin in beer can be different, most of them coming from the cereal raw material and a small part from the walls of the yeast cells, the two origins having different structural aspects [65]. In a review of Jayachandran et al. [34], in vitro, in vivo and clinical studies confirming the prebiotic action of these fibers are presented. In particular, β-glucans from barley and oats have been proven to reduce the levels of LDL and total cholesterol by modulating the microbial populations in the microbiota. Gut microbiota undergoes a shift in the direction of saccharolytic metabolism, producing SCFA, with the consequent decrease in protein metabolism products, such as p-cresyl sulfate (pCS) and indoxyl sulfate [66]. Β-glucans from oats have proven a greater power in stimulating the development of *Lactobacilli* and *Bifidobacterium* populations, compared to those from barley [67]. In a clinical study, 52 healthy subjects taking low doses of β-glucans from barley led to a significant increase in the number of *Bifidobacterium* [73,74]. Of course, the amount of β-glucans in beer is not large, most of it being degraded to glucose during the technological production processes. Moreover, the presence of β-glucans in barley generates technological problems. However, the remaining small amounts have the potential, along with other prebiotic elements in beer, to contribute to the modulation of the microbiota in a sanogenetic sense.

Arabinoxylans are another major component of beer’s fiber. Lynch et al. [69] used an extract of arabinoxylans from brewer’s spent grain to see the possible effects on the microbiota. Probiotic effects were obtained, consisting in the multiplication of lactobacilli populations by 2 times and bifidobacterial populations by 3.5 times. The same raw material, rich in arabinoxylans and β-glucans, was administered as a feed supplement to ruminants, causing the growth the activity of *Bifidobacterium*, *Enterococcus* and *Lactobacillus* [29]. In a study on human subjects, the effect of consuming bread enriched with oligosaccharides of arabinoxylans was followed [29]. Butyrate-producing colon bacteria and *Bifidobacterium faecalis* levels increased. In an in vitro study, arabinoxylans from maize species with various genotypes increased SCFA production, but in a genotype-dependent manner [70]. After drinking beer, an increase in the number of bacteria from the *Lachnospira* genus was found, which are usually SCFA producers with consecutive sanogenetic effects [30]. Different researchers emphasize the fact that the action on the microbiota is closely related to the degree of aggregation and branching of the arabinoxylans [71], their structure being very different depending on the source, its state of germination and the extraction methods.

It is difficult to generalize the effects on the microbiota because in vitro studies are not always reproduced in vivo, and most in vivo studies are based on supplements or products “designed” in the laboratory. In the real world, the action of arabinoxylans can be modified by the food matrix that they themselves influence, as in the case of beer. In vivo studies are required, using food products, possibly fortified, as long as the fortification does not make the product unacceptable from an organoleptic point of view [72]. Another element of variability is the subjects’ own microbiota that may or may not ferment such complex polysaccharides, some researchers finding very different effects depending on the geographic area from which the subjects came [73]. There are species from the microbiota genetically adapted (*Bacteroides*) to the digestion of complex arabinoxylans [98]. Moreover, when the excretion of SCFA is taken into account as an indicator of the in vivo action on the microbiota, it can be neglected that part of the SCFA has already been absorbed by the intestinal epithelium and thus the action of arabinoxylans can appear as negligible [99,100]. The fiber–microbiota relationship is bidirectional and both actors must be able to interact, so that possible positive effects can be highlighted.

From the multitude of contradictory results, a clear conclusion is that arabinoxylans and other soluble fibers present in beer are likely to positively influence the intestinal microbiota of consumers. Since arabinoxylans can bring organoleptic benefits to beer (increasing viscosity, foam stability), especially important in alcohol-free beers, it is assumed that an addition of arabinoxylans could be used intentionally, with collateral sanogenetic benefits [51,55]. However, producers must take into consideration the fact that consumers have a particular reluctance regarding the addition of additives and technological adjuvants in food, and beer is generally considered to be a drink based strictly on natural raw materials [74,101].

### 3.4. Melanoidins in Beer and Gut Microbiota

A type of substances that are often considered close to fiber because they have an extremely low digestibility [52] are the melanoidins. Their presence in beer originates from malted barley, they are formed in Maillard-type reactions. The structure of melanoidins in malt is very complex and is influenced by both the raw material, and the environmental conditions during the forming reaction [75,76]. Beer is dominated by a type of melanoidins called melanosaccharides, which are water-soluble substances [53]. Ingestion of melanoidins is variable in different populations, and beer contributes with relatively low levels compared to other foods [53]. Melanoidins in beer are valued especially for their organoleptic role, being involved in the color, texture and aroma of beer [28]. The amount of melanoidins in beer varies between 0.06 and 10.3 g/100 mL [77]. Rivero et al. [78] found, obviously, the highest amounts of melanoidin in dark beer, followed by blonde beer and Zhao et al. [80] described very large differences depending on the raw material and manufacturing methods. Alcohol-free beer had the lowest amounts (0.58 g/L). In recent years, however, numerous studies have focused on highlighting the influence of melanoidins on health, especially on their antioxidant, reducing capacity. Melanoidins in beer too have antioxidant roles [78,102]. However, because melanoidins reach the intestine largely undigested, it is assumed that they can also have a prebiotic role [80], being fermented by the intestinal microbiota [81]. The studies carried out in vitro and in vivo showed that the products of the Maillard reactions are decomposed by the intestinal flora, but so far there are no clear data on melanoidins as such. However, some studies argue that they have been shown to influence the intestinal flora, stimulating the growth of some local populations, fact that prove that bacteria can use the fermentation of melanoidins as a source of energy [103]. Other experiments support the fiber-like effect of melanoidins, but only after a long period of their administration, probably because the intestinal flora needs time to adapt to the digestion of substances with such a complex structure [104,105]. Moreover, the antioxidant influence of melanoidins on the digestive tract, demonstrated in various studies, could also be achieved through mechanisms mediated by the intestinal microbiota [106]. Although the studies on melanoidins as prebiotic fibers are few, they are not missing, and support the interaction with the gut microbiota. Aljahdali et al. administered to rats either malt without melanoidins (control group), or malt with increasing concentrations of melanoidins, for 3 weeks. The effects on the microbiota were important and differentiated, depending on the microbial species. The influence on SCFA production was also measured [107]. The populations of *Firmicutes* (*Dorea*, *Oscillibacter* and *Alisitpes*) decreased, but the first to respond were the populations of *Bacteroidetes*, then those of *Lactobacilli*, *Verrucomicrobia Acinobacteria* and *Proteobacteria (Parasutterella*), which increased. Two beneficial species, *Bifidobacterium* and *Akkermansia*, responded significantly only towards the end of the 3-week experiment, which supports the hypothesis of the need to adapt to melanoidins as an energy source. Stimulation of *Bifidobacterium* has been demonstrated in other studies, in which melanoidins from other food sources were used [79]. 

It should be noted that melanoidins also have an antibacterial role [82], being able to influence the microbiota in this way as well. They inhibit the development of pathogenic species, such as *Salmonella typhimurium*, *Staphylococcus aureus*, *Escherichia coli*, *Bacillus cereus* and *Pseudomonas aeruginosa* [83,108,109,110]. Melanoidins inhibited the ability of Streptococcus mutans to form adherent biofilms [107]. Of course, the intake of melanoidins through beer is not as high as the intake from other foods. Beer contribution is extremely variable, depending on the differences in beer consumption from one country to another and depending on the type of beer mostly consumed. However, the potential for interaction with the microbiota should not be neglected, and this process is still waiting to be defined more precisely through future studies.

## 4. Alcohol in Beer and Gut Microbiota

The content of alcohol is variable, and beer has the potential to be produced with minimal or no alcohol at all. However, widely consumed types of beer have generally around 5% alcohol. There are studies that discuss the link between alcohol and the gut–brain axis and the associated disorders such as alcoholic hepatitis, liver cirrhosis, anxiety, depression and impaired cognition performance that are a major cause of personal death and disability worldwide [111]. It is proven that the higher the percentage of alcohol in beer, the bigger the harmful impact of beer on gut microbiota and overall health. Nowadays, no level of alcohol intake can be considered safe. Alcohol modifies the gut microbiota composition and contribute to alcohol-induced oxidative stress, intestinal hyperpermeability to luminal bacterial products, and the subsequent development of alcoholic liver disease [112]. Alcohol is a factor that alters the normal function of the gut, it destroys the permeability of the intestinal membrane and by this it allows bacteria to enter the blood stream. A high constant alcohol intake has harmful effects on gut microbiota and the immune system [87,88,113,114,115]. Bacterial products that get through the non-intact intestinal barrier cause central inflammation; modify gut microbiota and impair enterohepatic circulation of bile acids; alcohol abuse causes shortage of vital nutrients such as thiamine [111]. The increased gut inflammation and intestinal hyperpermeability also induces endotoxemia, systemic inflammation and tissue damage/organ pathologies [112]. These arguments lead to the conclusion that only a minimal level of alcohol can be tolerated in functional beers and that the best alternative will be to fortify an alcohol-free beer.

## 5. Conclusions

The conclusions of this review are that beers (especially low or alcohol-free types) seem good candidates for future functional products and that there are many paths in which beer’s ingredients can influence in a positive way the human microbiota.

Combined actions of pre and probiotic factors can stimulate the proliferation or shift of the bacteria population towards a glycolytic one, normalizing its profile. Adding fiber, antioxidants and/or probiotics to beer are active ways to boosts its sanogenic properties but technological effects have to be balanced in such a way that fortification does not lower the acceptability of the product by consumers. Moreover, designing functional beers with a low or no alcoholic content will counteract the negative perception of beer consumption and will nullify the negative effects of alcohol. Even beer as it is, without any fortification, contributes at the intake of microbiota stimulating ingredients, that add to the sum of factors ingested by humans form a wholesome diet. However, we should aways take into consideration that no amount of alcohol is considered safe for health, and research has to target the development of functional beers without alcohol, that can be consumed at all ages and at of all groups of peoples. There is much research to be carried out in future but what it is known in this area up to now is encouraging for further positive results.

## Figures and Tables

**Table 1:** Types of microorganism from human gut microbiota.

Localization of Microbiota	Gut
Bacterial type	*Actinobacteria*
	*Bacteroidetes*
	*Firmicutes*
	*Lactobacillae*
	*Streptococci*
	*Enterobacteria*
Fungi type	*Candida*
	*Saccharomyces*
	*Malassezia*
	*Cladosporium*

**Table 2:** Summary of the cited papers according to their reference number.

Summary of Cited References	Reference, Name [Cited Number]
Composition of beer is complex, with many components having positive or negative action on human’s health.	Rosso et al. [1], Ambra et al. [4], Zugravu et al. [5], Sohrabvandi et al. [6], Díaz-Rubio et al. [7], Marcos et al. [11], Tucker et al. [12], Peng et al. [14], Fossi et al. [27], Fay et al. [28]
Beer’s interaction with microbiota is already proven in multiple studies and consists mostly by the re-equilibrating the composition of gut microbiota, with the stimulation of growth of saccharolytic microbes that generate SCFA.	Cecarini et al. [9], De Roos et al. [10], Redond et al. [13], Hou et al. [15], Thomas et al. [16], Costello et al. [17], Hillman et al. [18], Auchtung [19], Moissl-Eichinger et al. [21], La et al. [29], Zhang et al. [30]
Bacteria and other microorganisms can be present in beer, especially in non-pasteurized types. They can interact with gut microbiota, by means of a probiotic mechanism.	Ley et al. [22], Rinninella et al. [23], Flint et al. [24], Yatsunenko et al. [25], Das et al. [31], Bongaerts et al. [32], Broekaert et al. [33], Speers et al. [34], De Angelis et al. [35], La et al. [29]
Beer contains different antioxidants, especially polyphenols. Antioxidants from beer are influencing microbiota in a positive manner. Interaction with microbiota is bi-directional.	Takagak et al. [36], Ou et al. [37], Cardona et al. [38], Ghiselli et al. [39], Possemiers et al. [40], Marín et al. [41], Bartmańska et al. [42], Ozdal et al. [43], Hui et al. [44], Tomás-Barberán et al. [45], Hernández-Quiroz et al. [46] González-Zancada et al. [47], Khalil et al. [48], Pérez-Burillo et al. [49], Quesada-Molina et al. [50], Nguyen et al. [51], Millet et al. [52], Ekielski et al. [53]
Beer contains a multitude of non-digestible carbohydrates [fiber], thus interacting with gut microbiota by means of a prebiotic type of action.	Kanyer et al. [54], Li et al. [55], Li et al. [56], Kanauchi et al. [57], Goni et al. [58], Reid et al. [59], Diaz-Rubio et al. [60], Gibson et al. [61], Macfarlane et al. [62], Delcour et al. [63], Hughes et al. [64], Makelainen et al. [65], Jayachandran et al. [66], Cosola et al. [67], Shen et al. [68], De Angelis et al. [35], Lynch et al. [69], La et al. [29], Walton et al. [70], Zhang et al. [30], Louis et al. [71], Chen et al. [72], Zannini et al. [73], Li et al. [74], Salmen et al. [75]
Melanoidines are normal ingredients of beer, being present in different quantities. They can interact with microbiota by means of a prebiotic type of action.	Pérez-Jiménez et al. [76], Ekielski et al. [53], Fay et al. [28], Pastoriza et al. [77], Rivero et al. [78], Tagliazucchi et al. [79] Zhao et al. [80], Alves et al. [81], Pérez-Burillo et al. [49], Aljahdali et al. [82], Rufián-Henares et al. [83]
Beer spoilage can hinder beer fortification with different useful components.	Rodríguez-Saavedra et al. [84], Kordialik-Bogacka et al. [85]
Beer’s content of alcohol can hinder its use as functional food but can also influence positively the absorption of some components.	Wang et al. [3], Redond et al. [13], Engen et al. [86], Wang et al. [87], Calleja-Conde et al. [88]

## Data Availability

Not applicable.
